# Therapeutic potential of hepatocyte growth factor against cerebral ischemia (Review)

**DOI:** 10.3892/etm.2014.2133

**Published:** 2014-12-15

**Authors:** WEN ZENG, RONG JU, MENG MAO

**Affiliations:** 1 Department of Neonatology, Chengdu Women’s and Children’s Central Hospital, Chengdu, Sichuan 610031, P.R. China; 2 Department of Pediatrics, West China Second University Hospital, Sichuan University, Chengdu, Sichuan 610041, P.R. China; 3 Key Laboratory of Obstetric & Gynecologic and Pediatric Diseases and Birth Defects of Ministry of Education, Chengdu, Sichuan 610041, P.R. China

**Keywords:** hepatocyte growth factor, c-Met, cerebral ischemia, angiogenesis, antifibrosis, neurogenesis

## Abstract

The effective treatment for cerebral ischemia has not yet been established. Hepatocyte growth factor (HGF) is a potent pleiotropic cytokine that is involved in cell and tissue regeneration, including in the central nervous system. Studies have demonstrated that an exogenous administration of HGF protects brain tissue from ischemic damage. In response to binding to the receptor c-Met, HGF activates the downstream signaling pathways (including the phosphatidylinositol 3-kinase/Akt, Ras/MAPK and signal transducer and activator of transcription pathways) which leads to various cellular responses involved in angiogenesis, glial scar formation, anti-apoptosis and neurogenesis. The purpose of this review is to summarize the present understanding of the therapeutic potential of HGF in cerebral ischemia.

## 1. Introduction

Cerebral ischemia causes an irreversible and neurodegenerative disorder that may lead to progressive dementia and cognitive deterioration. However, no effective treatment has been established yet to prevent brain injury following ischemia.Hepatocyte growth factor (HGF), also referred to as Scatter factor, was first identified and purified from plasma and serum as a potent mitogen for hepatocytes in 1984 by Nakamura *et al* (1–3). Subsequently, HGF was identified in several other organs, including the lungs, kidneys and heart, as well as in blood vessels (4). In the 1990s, the wide distribution of HGF was identified in the central neural system (CNS) (5,6). HGF is now a well-known potent pleiotropic cytokine that is involved in mitogenesis, motogenesis, morphogenesis, angiogenesis and anti-apoptosis in a variety of cells, and tissue regeneration in several organs (7–9). HGF has been reported to improve the neurological sequelae by reducing the infarct volume following a stroke (10–12). This suggested that HGF should be one of the most potent growth factors for treating brain ischemia. In this review, we primarily focus on the role of HGF as a potential therapy for ischemic brain damage and the possible mechanisms.

## 2. HGF and its receptor c-Met

HGF was first identified as a mitogenic protein for rat hepatocytes in 1984 (13), and was thereafter purified from rat platelets, human plasma and rabbit plasma (14). In 1989, cDNA for human HGF was cloned and the primary structure of HGF was clarified, by which HGF was identified as a novel growth factor with unique structural characteristics (3). HGF is secreted as a single-chain, inactive polypeptide by mesenchymal cells and is cleaved to its active extracellular form by a number of proteases. The active HGF is a heterodimer composed of a 69-kDa α-chain and a 34-kDa β-chain. The α-chain contains an N-terminal hairpin domain followed by four kringle domains, and the β-chain contains a serine protease-like domain with no enzymatic activity (3,15).

The proto-oncogene product receptor tyrosine kinase c-Met is the only known receptor for HGF. The human Met (HGF receptor) gene is located on chromosome 7q21-q31. c-Met is synthesized as a 170-kDa glycosylated precursor protein that is cleaved into a 50-kDa α-chain and a 140-kDa β-chain that are linked by disulfide bonds (16).

In response to ligand (HGF) binding, c-Met undergoes autophosphorylation on two tyrosine residues (Y1234 and Y1235) within the activation loop of the tyrosine kinase domain, which regulate the intrinsic kinase activity of c-Met. Phosphorylation of Y1349 and Y1356 near the C-terminus of c-Met forms a multifunctional signal transducer docking site (Y1349VHVX3Y1356VNV) that binds a number of substrates containing Src homology-2 (SH2) domains, including growth factor receptor-bound protein 2 (Grb2), Gab1, phosphatidylinositol 3-kinase (PI3K), phospholipase C-γ (PLC-γ), Shp2 and Scr (17,18). This leads to the activation of downstream signaling pathways including the PI3K/Akt, Ras/MAPK and signal transducer and activator of transcription (STAT) pathways (19–21). Activation of the HGF/c-Met signaling pathway has been shown to lead to various cellular responses including proliferation, angiogenesis, wound healing, tissue regeneration, scattering, motility, invasion and branching morphogenesis.

## 3. HGF as a potential therapy for cerebral ischemia

### Angiogenesis

Angiogenesis was first described as a vital factor in tumor growth in 1971 (22) and then defined as the formation of new vessels sprouting from pre-existing capillaries in the pathological or physiological processes in adult tissue (23). It may be highly regulated by the action of growth factors, proteolytic enzymes or other extracellular matrix factors that stimulate the growth of endothelial cells. With the increased interest in angiogenesis and more in-depth research, it is considered that angiogenesis plays a significant role in minimizing tissue injury as the collateral blood flow supplies oxygen and energy substrate to the ischemic area. Therefore, the concept of therapeutic angiogenesis was proposed and became a new means of therapy, which is the clinical use of growth factors to enhance or promote the development of collateral blood vessels in ischemic tissue (24).

For a long time, attempts to alleviate ischemic cerebral injuries and ameliorate the prognosis have focused on ensuring or improving the survival of neurons, while ignoring the role of angiogenesis. However, the latter might be closely correlated with the survival of neurons following the ischemic insults. Krupinski *et al* (25) first reported that capillary density was increased in infarcted brain tissue of patients who had survived acute ischemic stroke for up to several weeks, indicating that increased angiogenesis is beneficial for longer survival of patients. Previous animal studies have revealed that inhibition of vascular endothelial cell proliferation promoted neural cell death, and that the application of proangiogenic regulator eased the ischemic injuries (26,27). Indeed, for treating limb ischemia and myocardial infarction, proangiogenic regulators became a new means of therapy. Angiogenesis should be a potent therapy for stroke patients through increasing the cerebral blood flow. Other studies also demonstrated that vascular endothelial growth factor (VEGF), a notable proangiogenic factor (28), increases vascular density, reduces brain damage and improves neurological deficits (29–32), suggesting that angiogenic therapy may be helpful for ischemic brain injury.

As a potent angiogenic molecule, HGF mediates angiogenesis primarily through direct actions on vascular endothelial cells. Studies have demonstrated that HGF and c-Met are expressed and functional in vascular endothelial cells of various origins, including neuromicrovascular endothelial cells (33). Shang *et al* observed that HGF significantly amplified the angiogenesis following middle cerebral artery occlusion (34). Date *et al* (35,36) also reported that HGF could prevent the learning and memory dysfunction induced by cerebral ischemia by protecting the endothelial cells against injury.

The molecular mechanisms of the angiogenic activity of HGF may be strongly associated with the E-twenty-six (ETS) pathway, since the ETS family plays a significant role in regulating gene expression in response to the multiple developmental and mitogenic signals (37,38). By activating the specific receptor, c-Met, HGF also induces DNA synthesis and proliferation of vascular endothelial cells through MAPK/ERK and STAT3 pathway activation (39,40).

In addition to the proliferative effects, HGF also protects endothelial cells against apoptosis or cell death induced by various detrimental insults, including hypoxia and serum deprivation. However, the signal pathways that mediate the protective effects are not fully known. Ma *et al* (41) revealed that the MAPK/ERK and Akt pathway may mediate the HGF-induced survival of endothelial cells. Other experiments indicate that HGF protects endothelial cells against hypoxic injury associated with inhibition of p38 MAPK and Bid/Bax as well as increased expression of Bcl-2 or Bcl-xl (42,43).

It is known that matrix degradation and remodeling are indispensable in angiogenesis, and allow endothelial cell migration and invasion (44,45). HGF induces or upregulates the expression and synthesis of matrix metalloproteinases (MMPs) and urokinase-type plasminogen activator by vascular endothelial cells, and accelerates endothelial cell invasion into the extracellular matrix during angiogenesis (46,47). HGF also stimulates dissociation and migration of vascular endothelial cells, which may occur through the modulating action of HGF on the vascular endothelial cadherin (48).

In addition, HGF regulates angiogenesis through interacting with other well-known angiogenic regulators. It has been shown that HGF induces VEGF expression at both the mRNA and protein levels, which might be regulated by MAKP, PI3K, PKC and Sp1, a modulator of the VEGF promoter (40,48). Further studies identified that essential transcription factor ets-1 was upregulated by HGF and contributed to HGF-induced VEGF expression (38,49). HGF and VEGF play synergistic effects in promoting vascular endothelial cell survival with augmented expression of the anti-apoptotic genes Bcl-2 and A1 (48). Moreover, HGF is capable of downregulating the expression of thrombospondin 1, which negatively regulates angiogenesis (50,51).

Furthermore, compared with other angiogenic regulators including VEGF, HGF has noted advantages in promoting angiogenesis, as follows: a) it does not disrupt the blood brain barrier (BBB); b) it does not increase cerebral edema; c) it does not cause vascular inflammation; and d) it has anti-thrombosis ability (35,36,52,53). VEGF is a major mediator of angiogenesis as well as being a strong vascular permeability factor and a notable stroke-related pathogenic factor for the formation of brain edema (54–56), which limits its therapeutic applications. HGF avoids these disadvantages. It is suggested that HGF-mediated prevention of endothelial cell injury and maintenance of the tight junctional proteins in the endothelial cells may be a possible mechanism for the protective effect of HGF against the disruption of the BBB and the prevention of cerebral edema (36).

### Anti-apoptosis and neurogenetic effects

Since mature neurons cannot duplicate, it is essential to maintain their survival to improve the outcome of cerebral ischemia. The roles of excitatory amino acid receptor activation, calcium overload, nitric oxide and oxidative stress are well established in the pathogenesis of ischemic brain damage (57). Studies have reported that HGF notably decreases the infarct volume of ischemic brain tissue and protects neurons from death caused by N-methyl-D-aspartate excitotoxicity (11), and it also prevents neuron death by inhibition of apoptosis through the blockade of bax translocation from the cytoplasm to the nucleus (58,59). In addition, HGF stimulates ERK1/2, PI3K/Akt and STAT3 activity in neurons, and then induces the transcription of neuroprotective genes, including bcl-2 and Bcl-xl, protecting neurons from apoptosis following ischemia (39,60–62).

In recent years, there has been a growing interest in the therapeutic potential of stem cells. Stem cells are multipotent and self-renewing cells, so it is believed that they may be beneficial to the outcome of cerebral stroke. There is evidence revealing that transplantation of neural stem cells (NSCs) or mesenchymal stem cells (MSCs) decreases the infarcted area and improves functional outcomes (63–66). HGF promotes the proliferation, differentiation and migration of NSCs and MSCs (67–69). However, the mechanisms involved are still not completely understood. It has been reported that HGF induces the activation of its downstream effectors ERK1/2, p38MAPK and PI3K/Akt, which contribute to the effects of modulating the migration, proliferation and differentiation of NSCs and MSCs, while the regulation is abrogated by specific inhibitors (68–72).

### Antifibrosis

Ischemia induces tissue damage to the CNS and activates astrocytes, leading to reactive gliosis, which causes glial scar formation (73). A glial scar presents as a rubbery, tenacious growth-blocking membrane, which consists predominately of reactive astrocytes and chondroitin sulfate proteoglycans, including neurocan and phosphacan (74,75). Although studies indicate that glial scarring serves to repair the BBB, prevent an excessive inflammatory response and limit cellular degeneration following injury, it also has the disadvantage of inhibiting neuronal and axonal regeneration, causing failure in the structural and functional reconstruction of the CNS following injury (75–79). Therefore, inhibiting glial scar formation is a critical issue for nerve regeneration and functional reconstruction.

As a multifunctional cytokine, it was suggested by Ha *et al* (80) that HGF prevents pathological scar formation *in vivo* and *in vitro*. Another study revealed that HGF decreases glial scar formation and scar thickness of the brain pia mater following transient middle cerebral artery occlusion, indicating that HGF is beneficial for ischemic injury due to its antifibrotic ability (34). A possible mechanism is that HGF markedly inhibits the proliferation and migration of astrocytes in the formation process of glial scarring by the sphingosine-1-phosphate pathway, which is closely related to cell proliferation (81).

Aside from the proliferation and activation of astrocytes, several other factors are also associated with the formation of glial scarring, including transforming growth factor-β (TGF-β) and extracellular matrix components. Previous studies suggest that HGF plays antifibrotic roles by regulating proteoglycan synthesis. When astrocytes are activated, four classes of proteoglycans are produced by astrocytes, which are closely related to glial scar formation (73,74,82). TGF-β1 is a potent fibrogenic protein that has been shown to significantly increase the production of proteoglycans by astrocytes and cause severe astrogliosis (83,84). However, Jeong *et al* revealed that HGF completely blocked secretion of TGF-β1 from activated astrocytes (85). After binding to c-Met, HGF upregulates the activity and protein expression of est-1 (38). est-1 has a DNA-binding domain and activates transcription of genes encoding uPA and various metalloproteases (e.g., MMP-1 and MMP-9) (86). Via the est-1 pathway, HGF plays its role in decreasing glial scar formation.

## 4. Challenges

The clinical use of HGF is quite limited at present for a number of reasons, including lack of effective administration methods and adverse effects. We will discuss these below.

### Administration methods

A notable breakthrough has been made in using HGF in the therapy for limb ischemia, which is at the clinical trial stage (87). However, the administration of HGF as a recombinant protein for CNS disorders is hindered by a number of issues, including the short serum half-life and poor access to the CNS by the systemic route due to the presence of BBB.

Gene therapy may solve the issue of degradation, but the safety and efficiency of the gene carrier must be ensured. Retroviruses are one of the widely used gene carriers, and can integrate the gene into the chromosomes of the target cells (88–91). However, clinical use is limited due to the potential rise of a neoplasm with a retrovirus-based vector (92).

Considering the safety of gene therapies, certain scientists have proposed a new solution: transferring the virus vector into the host cells (e.g., MSCs) and then transferring the cells into the injured organs (93–95). As reported, MSCs have protective effects in cerebral ischemia (64,93). After being transfected with vector encoding HGF gene, increasing the expression of HGF, the protective effects are enhanced (85,96,97). This method also avoids the possible detriment of virus vector and decreases immunity reactions. However, this therapeutic method is still tested on animals and needs considerable research and effort for clinical practice.

### Adverse effects of tumorigenesis

Previous studies have demonstrated that HGF plays a role in tumorigenesis through its capability to promote angiogenesis and mitogenesis (98–101). Therefore, if HGF were used to treat ischemic brain injury, the issue of how to decrease or eliminate the risk of tumorigenesis must be taken into consideration. This issue could possibly be solved by selecting the optimal concentration and time point of medication.

## 5. Conclusion

Overall, as a growth factor, HGF has therapeutic potential against cerebral ischemia. Binding to the receptor c-Met, downstream signaling pathways are phosphorylated and activated, including the PI3K/Akt, Ras/MAPK and STAT pathways, then HGF is capable of regulating angiogenesis, glial scar formation, neurogenesis and anti-apoptosis, protecting the brain from ischemic insults. Although certain obstacles remain before clinical application of HGF can be achieved, we are of the opinion that through the deepening research these issues will be overcome, bringing benefit to patients with cerebral ischemia.
